# Association Between Females and Males in the Prevalence of Depression in the Gulf Cooperation Council (GCC) Countries

**DOI:** 10.1192/bjo.2024.176

**Published:** 2024-08-01

**Authors:** Mitha Al Balushi, Syed Javaid, Fatima Al Maskari, Shamil Wanigaratne, Amar Ahmad

**Affiliations:** 1 Public Health Research Center, New York University-Abu Dhabi, Abu Dhabi, UAE; 2 Institute of Public Health, College of Medicine and Health Sciences, United Arab Emirates University, Alain, United Arab Emirates, Abu Dhabi, UAE; 3 Department of Psychiatry and Behavioral Sciences, College of Medicine and Health Sciences, Abu Dhabi, UAE; 4 King's College London, London, United Kingdom

## Abstract

**Aims:**

In most populations, the prevalence of depression is more significant in women than in men. Nonetheless, the degree of gender disparity varies significantly across countries. The aim of this study is to consider the role of gender inequality in explaining these differences in the Gulf Cooperation Council (GCC) countries.

**Methods:**

Data on the ecological prevalence of depression (males versus females) from 1990–2019 from the GCC countries were downloaded from Our World in Data and included in the statistical analysis. A mixed-effects linear model was used to examine the association between males and females, i.e. females regress on males. Year and country variables were used as random effect variables.

**Results:**

The prevalence of depression in the GCC countries shows a gender-specific pattern with a higher prevalence in females than in males 1.218 (95% CI: 1.149–1.285), p-value < 0.001. Higher levels of depression between men and women were observed in Kuwait and Saudi Arabia compared with the other four countries. The lowest depression prevalence was observed in the United Arab Emirates.

**Conclusion:**

The pattern of depression in the GCC countries is based on gender. However, the association between global measures of gender inequality and the gender gap in depression may depend on how the level of depression is measured. More research is needed to investigate the mechanisms that underlie the gendered nature of depression prevalence.

